# Self-assembled polymeric nanocarrier-mediated co-delivery of metformin and doxorubicin for melanoma therapy

**DOI:** 10.1080/10717544.2021.1898703

**Published:** 2021-03-17

**Authors:** Mingming Song, Wentao Xia, Zixuan Tao, Bin Zhu, Wenxiang Zhang, Chang Liu, Siyu Chen

**Affiliations:** State Key Laboratory of Natural Medicines and School of Life Science and Technology, China Pharmaceutical University, Nanjing, China

**Keywords:** Drug delivery, melanoma, combination therapy, nanoparticles, metformin, doxorubicin, PANoptosis

## Abstract

Malignant melanoma is a life-threatening form of skin cancer with a low response rate to single-agent chemotherapy. Although combined therapies of metformin (MET) and doxorubicin (DOX) are effective in treating a variety of cancers, including breast cancer, their different physicochemical properties and administration routines reduce the effective co-accumulation of both drugs in tumors. Nanoparticles (NPs) have been demonstrated to potentially improve drug delivery efficiency in cancer therapy of, for example, liver and lung cancers. Hence, in this study, we prepared pH-sensitive, biocompatible, tumor-targeting NPs based on the conjugation of biomaterials, including sodium alginate, cholesterol, and folic acid (FCA). As expected, since cholesterol and folic acid are two essentials, but insufficient, substrates for melanoma growth, we observed that the FCA NPs specifically and highly effectively accumulated in xenograft melanoma tumors. Taking advantage of the FCA NP system, we successfully co-delivered a combination of MET and DOX into melanoma tumors to trigger pyroptosis, apoptosis, and necroptosis (PANoptosis) of the melanoma cells, thus blocking melanoma progression. Combined, the establishment of such an FCA NP system provides a promising vector for effective drug delivery into melanoma and increases the possibility and efficiency of drug combinations for cancer treatment.

## 1. Introduction

Malignant melanoma, the major fatal form of skin cancer, accounts for more than 90% of all skin cancer-related deaths (Miller et al., 2020; Siegel et al., 2020). Although genetic susceptibility and skin pigmentation are dominant risk factors, environmental factors such as occupation and latitude have been identified to affect the morbidity and motility of melanoma (Turner et al., 2020). An estimated 100,350 new cases of skin melanoma will be diagnosed at the end of 2020, which will lead to a nearly 6.8% incidence of death (Siegel et al., 2020). Hence, malignant skin melanoma greatly threatens public health and causes a heavy burden on all social economies.

Currently, there are several approaches available for the treatment of melanoma, including chemotherapy, surgery, biological therapy, radiotherapy, and immunotherapy(Cullen et al., 2020; Dafni et al., 2019; Kim et al., 2019). Among these, conventional drug-based therapeutics are severely limited owing to their lack of therapeutic efficacy, high toxicity to healthy tissues, and nonspecificity toward cancer cells (Sharma et al., 2017; Nakajima et al., 2020). Notably, the combined application of MET (Wang et al., 2020a), photodynamic-immunotherapy (Hu et al., 2021) and chemotherapy (e.g. DOX) (Ci et al., 2020; Wang et al., 2020b; Fu et al., 2021) can elevate their anti-tumor abilities, reduce treatment doses, and circumvent side effects. However, clinical limitations of MET still exist because of its short half-life and weak bioavailability, and high doses of this drug cause unexpected side effects (e.g. lactic acidosis and liver dysfunction) (Li et al., 2019, 2020; Cheng et al., 2019). In addition, the differences in the drug administration routines followed for hydrophobic DOX and hydrophilic MET result in differences in their pharmacodynamics, causing time discrepancies in drug metabolism, and decrease the effective co-accumulation of the two drugs in tumor targeting (Da Silva et al., 2019; Wang et al., 2020c; Sun et al., 2020). Hence, a safe and efficient approach is required for the simultaneous co-delivery of MET and DOX into melanoma for treatment.

The application of nanotechnology in cancer treatments has rapidly developed, and addresses several limitations of traditional drug delivery systems, such as nonspecific biodistribution and targeting, lack of water solubility, and poor oral bioavailability (Ling et al., 2021; Hasani-Sadrabadi et al., 2020; Rogalla et al., 2019; Jain & Stylianopoulos 2010). To improve the biological distribution of cancer drugs, optimally sized nanocarriers with specific surface characteristics have been designed, allowing the drug to remain in the vasculature via passive accumulation at tumor sites based on the enhanced permeability and retention effect (EPR) (Tee et al., 2019; He et al., 2019). Furthermore, nanocarriers can improve drug bioavailability by sustaining drug release and avoiding reticuloendothelial system-driven clearance (Kumar Giri et al., 2016; Oh et al., 2020; Mohamed et al., 2021). A variety of nanoparticles have been developed to deliver effective drugs or functional nucleotides for melanoma treatment, thus suppressing melanoma tumor progression (Wei et al., 2020; Tang et al., 2017). These nanocarriers are composed of metal and inorganic particles, including iron, gold, and silica, ignoring systemic immune recognition of exogenous materials and leading to an unpredicted immune response, which may aggravate melanoma progression (Palanikumar et al., 2020; Kuang et al., 2020; Benyettou et al., 2020; Bagheri et al., 2018). Therefore, establishing an immune-irresponsive nanosystem to deliver functional drugs into melanoma cells may be valuable for the treatment of melanoma cancer.

**Scheme 1.**
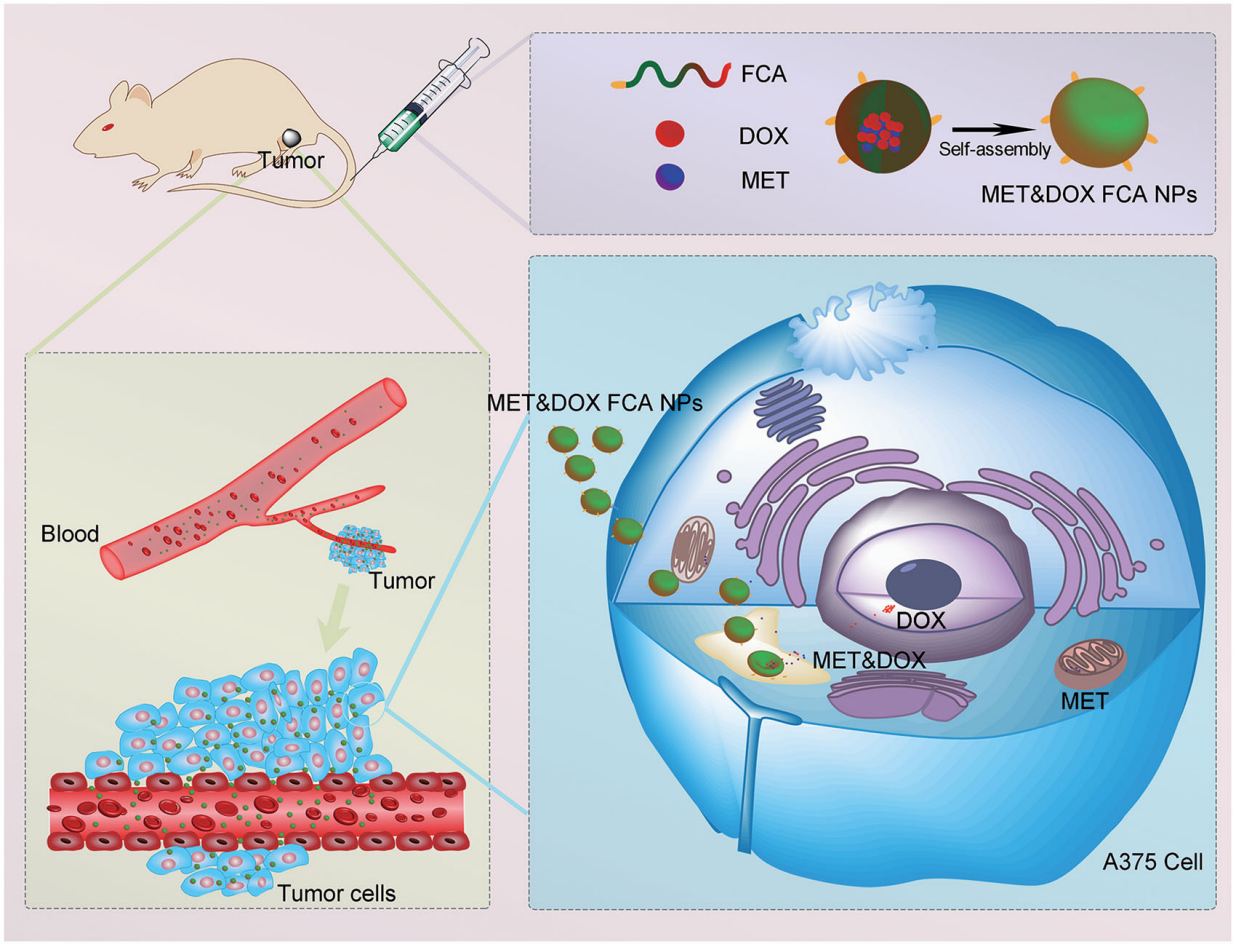
Schematic illustration of the self-assembled polymeric nanocarrier-mediated co-delivery of metformin and doxorubicin for melanoma therapy.

In the present study, we prepared pH-sensitive, biocompatible, tumor-targeting nanoparticles, folic acid-cholesterol-sodium alginate NPs (FCA NPs), based on the combination of sodium alginate with cholesterol and folic acid. Among these, sodium alginate, a linear and anionic polysaccharide consisting of two 1,4-linked hexuronic acid residues, forms the hydrophilic shell of FCA NPs. This material has been widely used for cancer drug delivery treatments owing to its biocompatibility, low cytotoxicity, and ability to self-assemble into nanoparticles under mild conditions (Zheng et al., 2020). In addition, cholesterol, one of the endogenous substances involved in various biological processes (Hu et al., 2017), functions as the hydrophobic core of FCA NPs. Additionally, since cholesterol is an endogenous metabolite within the body, it is host-friendly and would circumvent host immune responses. Last but not least, to achieve specific targeting efficiency, molecular targeting agents, such as antibodies, peptides, and folic acid, have been grafted onto nanodrug delivery systems (Liu et al., 2019). Among which, folic acid is an essential component in DNA synthesis, repair, and methylation, as well as amino acid and RNA metabolism, and plays a vital role in cell growth and division (Angelopoulou et al., 2019). Given that cancer cells, including melanoma cells, express high levels of folate receptors, and utilize more folate than normal cells to maintain their uncontrollable growth (Musalli et al., 2020), the folic acid/folate receptor axis is considered as a molecular target for cancer treatment. Based on these aforementioned benefits, we grafted folic acid onto cholesterol-sodium alginate (CA) to deliver functional drugs into folate receptor-overexpressing melanoma cancer cells. Taking advantage of this nanosystem, we successfully co-delivered MET and DOX into melanoma tumors and found that our FCA-NPs increased the anti-tumor effects of the combination of MET&DOX (Scheme 1). Collectively, our findings present a promising nanocarrier to improve the therapeutic effects of drug combination-based tumor therapy.

## 2. Materials and methods

### 2.1. Materials

Sodium alginate, folic acid, and cholesterol were purchased from Sangon Biotech Co., Ltd. (Shanghai, China). MET, filipin, N,N'-dicyclohexylcarbodiimide (DCC), 4-(dimethyl-amino) pyridine (DMAP), (N1-(ethylimino-methylene)-N3, N3-dimethylpropane-1,3-diamine (EDC), N-hydroxysuccinimide (NHS), and dimethyl sulfoxide (DMSO) were obtained from Sigma-Aldrich Company (St. Louis, MO, USA). Doxorubicin, chlorpromazine hydrochloride, and EIPA were obtained from Shanghai Aladdin Bio-Chem Technology Co. Ltd. (Shanghai, China). All other chemicals and reagents used in this study were of analytical grade.

### 2.2. Cells and animals

All cells were obtained from the American Type Culture Collection (ATCC). Human A375 and SK-MEL-28 cells were cultured in Dulbecco's modified Eagle's medium (DMEM, Gibco, Waltham, USA) supplemented with 10% fetal bovine serum (FBS, Sciencell, San Diego, USA) with 1% antibiotics (penicillin streptomycin, Sigma-Aldrich, St. Louis, MO, USA) at 37 °C in a humidified atmosphere containing 5% CO_2_ (Zhang et al., 2019; Chen et al., 2020). Male C57BL/6J mice (8-weeks old) and male BALB/C-NU mice (5-weeks old) were purchased from the Model Animal Research Center of Nanjing University (Nanjing, China). All animal procedures in this investigation conformed to the Guide for the Care and Use of Laboratory Animals published by the US National Institutes of Health (NIH publication No. 85–23, revised 1996) and the approved regulations set by the Laboratory Animal Care Committee at China Pharmaceutical University (Permit number SYXK-2016–0011). All mice were maintained in a 12 h light-dark cycle in a temperature- and humidity-controlled environment (Zhang et al., 2020).

### 2.3. Synthesis and characterization of the FCA conjugates

The synthetic procedure for the CA was adapted from a previously reported method with minor modifications (Song et al., 2017; Wang et al., 2015). Briefly, 1.0 g sodium alginate, 100 mg of DCC, and 125 mg DMAP were added to 50 mL deionized water to activate the carboxyl groups on sodium alginate. Then, 1.0 g of cholesterol was added to the mixture, with stirring, and allowed to react for 24 h at room temperature. Finally, 25 mL ethanol was added to the reaction mixture to precipitate the product, which was separated by centrifugation. After thorough washing with absolute ethanol, the products were neutralized by adding a 1.5% mixture of Na_2_CO_3_ and NaHCO_3_. Subsequently, the solution was dialyzed against distilled water for 3 days and lyophilized to obtain the pure CA product according to a previously reported method. The FCA conjugate was synthesized according to a previously reported method. First, folic acid was activated with EDC and NHS at a molar ratio of 1:1:1 in 15 mL DMSO. Next, the activated folic acid was slowly added to a 1.0% (*w*/*v*) solution of CA. The mixture was stirred overnight at room temperature in the dark. The reactant mixture was then filtered and dialyzed against distilled water for 3 days using a dialysis tube (molecular cutoff: 3500 Da) to remove the free folic acid. Finally, the resultant FCA was isolated by lyophilization. Characterization of the FCA was performed using ^1^H NMR (AVANOE, Bruker, Rheinstetten, Germany).

### 2.4. Preparation and characterization of the FCA NPs

Freeze-dried FCA powder (5 mg) was dissolved in 5 mL ultrapure water (1 mg/mL) with gentle shaking at 37 °C for 1 h, followed by sonication using an ultra-sonicated bath at 100 W for 7 min. The solution of self-assembled NPs was then filtered through a 0.8 μm Millipore filter to remove any dust and impurities. The morphology of the self-assembled FCA NPs was observed using transmission electron microscopy (TEM). The size, PDI, and zeta potential of the prepared NPs were determined using a Malvern Zetasizer system (ZEN3690, Malvern Instruments Limited, Malvern Worcestershire, UK).

### 2.5. Stability of the nanocarrier

To evaluate the stability of the nanocarrier under physiological conditions, the FCA NPs were dispersed in the PBS (pH 5.0 or pH 7.4) and PBS (pH 7.4) containing 10% FBS(Zheng et al., 2019). The particle size was measured at 0 and 48 h using a Malvern Zetasizer system at 25 °C. The FCA NP solutions were collected after 48 h of incubation and the morphology of the NPs observed by TEM. Next, the pharmacological safety of the FCA NPs was evaluated using a hemolysis assay. In brief, blood samples were obtained from the male C57BL/6J mice and the erythrocytes were separated by centrifuging the blood at 1500 rpm for 15 min. To prepare pure red blood cells (RBCs), the RBCs were washed thrice with normal saline at a ratio of 1:20. The CA and FCA NPs were subsequently diluted to different concentrations and incubated with the RBCs for 30 min at 37 °C. Subsequently, the RBCs were separated by centrifugation (4000 rpm for 10 min), and images were acquired for visual comparison.

### 2.6. Cellular distribution and uptake of the FCA NPs

Cy5, a fluorescent dye, was added during the synthesis of the FCA NPs. To study the cellular uptake of Cy5-labeled FCA NPs, A357 cells were seeded on a confocal dish containing 4 cm^2^ slides at a density of 1.0 × 10^5^ cells/mL and incubated overnight. Cy5 and Cy5-labeled FCA NPs were added to replace the media in each confocal dish. After further incubation for 1, 4, 6, and 12 h, the cells were washed to remove non-internalized particles, fixed with 4% paraformaldehyde, and the lysosomes stained with Lyso-Tracker Green, followed by incubation with 4′,6-diamidino-2-phenylindole (DAPI) for another 5 min. Cellular uptake was observed using confocal laser scanning microscopy (CLSM). The cellular uptake efficiency of the A375 cells was determined using flow cytometry. The A357 cells were seeded in 12-well plates at a density of 1 × 10^3^ cells/well and allowed to grow and proliferate as a monolayer under standard conditions for 24 h. The culture media were discarded and replaced with fresh medium supplemented with Cy5-labeled FCA NPs at a concentration of 250 μg/mL and incubated for 12 h. The cells were collected with 25% ethylene diamine tetraacetic acid (EDTA) trypsin, washed twice, and resuspended in PBS. Flow cytometry was used to measure the fluorescence intensity in the cells. To further identify the cellular uptake mechanism of the FCA NPs (Islam et al., 2018), A357 cells were seeded on confocal dishes containing 4 cm^2^ slides at a density of 1.0 × 10^5^ cells/mL and incubated overnight. Next, the culture media were discarded and replaced with fresh media containing inhibitors (filipin, 1 μg/mL), chlorpromazine (10 μg/mL), EIPA (10 μg/mL), and Baf A1 (200 nM). After incubation for 2 h, the culture media were removed and replaced with fresh media containing Cy5-labeled FCA NPs followed by incubation for 12 h. Finally, the cells were fixed and the red fluorescence of Cy5 obtained using CLSM (LSM700, Carl Zeiss, Oberkochen, Germany) and processed using ZEN imaging software (Carl Zeiss, Oberkochen, Germany).

### 2.7. *In vivo* biodistribution

To establish the xenograft model, 2.5 × 10^6^ A375 cells were inoculated subcutaneously into the right flanks of nude mice. When the tumor size reached approximately 100–150 mm^3^, 200 μL Cy5 or Cy5-labeled FCA NPs (Cy5 concentration: 30 μg/mL) were *i.v.* injected. *Ex vivo* imaging was performed 24 h after administration. Next, the major organs, including the heart, liver, spleen, lung, kidney, and tumor, were harvested from the euthanized mice and examined using an IVIS Lumina III In Vivo Imaging System (Perkin Elmer, Alameda, USA) to visualize the distribution of the fluorescent signals of Cy5 (Cy5 and Cy5-labeled FCA NPs).

### 2.8. Encapsulation efficiency (EE) and loading efficiency (LE) of the FCA NP-loaded drugs

FCA NP-loaded MET and DOX was prepared using a similar approach to that described in Section preparation of the FCA NPs. First, 5 mg of freeze-dried FCA powder was dissolved in 5 mL ultrapure water (1 mg/mL). In the typical one-step self-assembly of the NPs, MET (5–20 mg) or DOX (0.1–1 mg) was dissolved in the FCA solution and mixed for 6 h on a shaker. Then, the resulting solution was ultra-sonicated at 100 W for 7 min. Subsequently, the FCA NP-loaded MET and DOX solutions were kept at room temperature for 4 h and centrifuged at 12,000*g* for 30 min to remove the supernatant and obtain the sediment. After the drug-loading experiments, the EE and LC for MET (DOX) in the FCA NPs were calculated by determining the concentration of MET (DOX) in the supernatant. The experiments were conducted in triplicate, and the following formulas were used:

EE% = 100 × (Total amount of MET (DOX)−Amount of unencapsulated MET (DOX))/Total amount of MET (DOX)

LE% = 100 × (Total amount of MET (DOX)−Amount of unencapsulated MET (DOX))/Total weight of NPs

### 2.9. *In vitro* release examination

The *in vitro* drug release tests were conducted using a dynamic dialysis method; a membrane with a molecular weight cutoff of 2000 Da was used. Briefly, 1 mL of FCA NP-loaded MET and FCA NP-loaded DOX solutions were placed in a dialysis bag. The solution was then incubated in a 5 mL release medium (pH 5.0 and pH 7.4, PBS) at 37 °C, followed by shaking at 100 rpm. At different time intervals, 100 μL of the release medium was collected and supplemented with equal volumes of fresh release media. The amounts of MET and DOX released were measured using UV-vis spectroscopy (MET: 233 nm, DOX: 488 nm) (Lambda 25, Perkin Elmer, Waltham, USA).

### 2.10. Cytotoxicity assay

The 24 h cytotoxic effect of the FCA NPs on human A375 or SK-MEL-28 cells was assessed using a CCK-8 assay. Briefly, 5 × 10^3^ cells were seeded onto a 96-well plate and incubated for 24 h, followed by treatment with indicated concentrations of the different drugs for another 24 h. Subsequently, 10 μL of WST-8 reagent (Jiancheng Institute of Biotechnology, Nanjing, China) was added to each well and incubated at 37 °C for another 2 h. Finally, a microplate reader was used to measure the absorbance at 450 nm.

### 2.11. Serological and complete blood count (CBC) analyses

Serum samples were collected in a centrifuge tube and centrifuged at 4000 rpm for 10 min at 4 °C. The serum levels of aspartate transaminase (AST), alanine aminotransferase (ALT), blood urea nitrogen (BUN) and creatinine were determined using commercial kits (Jiancheng Institute of Biotechnology, Nanjing, China). For the CBC analysis, blood samples were collected in an anticoagulant tube and examined using a Fully Auto Hematology Analyzer (BC 2800-Vet, Mindray, Shenzhen, China).

### 2.12. *In vivo* antitumor efficacy

The human A375 melanoma model was established by subcutaneously injecting 2.5 × 10^6^ human A375 cells into the right posterior flank of BALB/C-NU mice. When the tumor volume reached 50 mm^3^, the mice were randomly divided into seven groups and treated with PBS (control group) and the drugs (MET, DOX, MET&DOX, FCA NP-loaded MET, FCA NP-loaded DOX, and FCA NP-loaded MET&DOX). The tumor volumes and body weight of the mice were measured every three days for 16 days. The tumor volume was calculated using the formula: tumor volume = length × width × width/2. After the 16-day drug treatment, the mice were sacrificed. Major organs, sera, and tumors were collected for further analysis.

### 2.13. Flow cytometry analysis

The cells were plated in 6-well plates and cultured at 37 °C for 24 h. The human A375 and SK-MEL-28 cells were prepared, fixed, and incubated with Annexin-V APC/7-AAD for cell apoptotic analysis according to the manufacturer’s instructions. Flow cytometry analysis was performed using a BD LSRFortessa (BD Biosciences, San Jose, USA).

### 2.14. Tunel assay

The TUNEL method was used to label the 3′-end of fragmented DNA from the apoptotic human A375 and SK-Mel-28 cells. Cells were collected from the different groups, fixed with 4% paraformaldehyde, and stained with the one-step TUNEL apoptosis assay kit (Beyotime Biotechnology, Shanghai, China) according to the manufacturer’s instructions. Furthermore, the tumor tissues were stained using the same procedure after embedding and dehydration. The number of positive cells was then assessed using images obtained by fluorescence microscopy (Nikon fluorescence microscope, ECLIPSE, Ts2R-FL, Tokyo, Japan).

### 2.15. Histological analyses

Fresh samples were fixed in a 4% paraformaldehyde solution for 24 h in situ, processed for paraffin embedding, and cut into 5 μm transverse sections for routine H&E staining. For immunohistochemical (IHC) staining, the slides were brought to room temperature and then incubated with individual primary antibodies against Ki-67 (Cat. No. GB111141, 1:1000 dilution, Servicebio, Wuhan, China), GSDMD (Cat. No. SC-81868, 1:50 dilution, Santa Cruz Biotechnology, Inc., Santa Cruz, USA), Caspase-7 (Cat. No. 27155-1-AP, 1:1000 dilution, Proteintech, Chicago, USA), and MLKL (Cat. No. 21066-1-AP, 1:1000 dilution, Proteintech, Chicago, USA) at 4 °C overnight. The slides were incubated with appropriate horseradish peroxidase (HRP)-conjugated secondary antibodies at room temperature for 30 min. Finally, the slides were incubated with 3,3-diaminobenzidine (DAB) for visualization using a NanoZoomer S210 (NanoZoomer 2.0 RS, Hamamatsu Japan).

### 2.16. Western blots

The human A375 and SK-MEL-28 cells were lysed in radioimmunoprecipitation assay buffer. The cellular proteins were extracted from cold environments. Equal amounts of protein were loaded and separated by 10% SDS-PAGE and transferred onto a polyvinylidene difluoride membrane (Millipore, Bedford, USA). The membranes were blocked with 5% nonfat milk in PBS, and the primary antibodies were added and incubated overnight. Bound antibodies were visualized using HRP-conjugated secondary antibodies. Quantitative analysis was performed using AlphaEaseFC software (AlphaInnotech, San Leando, CA, USA). The antibody against GSDMD (Cat. No. SC-81868, 1:200 dilution) was purchased from Santa Cruz Biotechnology, Inc. (Santa Cruz, CA, USA). Antibodies against CASPASE-7 (Cat. No. 27155-1-AP, 1:1000 dilution, Proteintech, Chicago, USA), and MLKL (Cat. No. 21066-1-AP, 1:1000 dilution) were purchased from Proteintech (Chicago, IL, USA). The antibody against β-actin (Cat. No. BS6007MH, 1:1000 dilution) was purchased from Bioworld Technology (Nanjing, China).

### 2.17. Statistical analysis

All results are expressed as the mean ± standard deviation (SD). Statistical significance of differences was analyzed using GraphPad Prism 7 software (GraphPad Software, San Diego, USA). Normality of the quantitative data was first analyzed using SPSS (version 19.0, IBM, Armonk, USA). *P* values were calculated using one-way ANOVA followed by Tukey’s multiple comparison test. A *p-*value less than 0.05 was considered as statistically significant.

## 3 Results and discussion

### 3.1. Preparation and characterization of the FCA NPs

**Figure 1.**
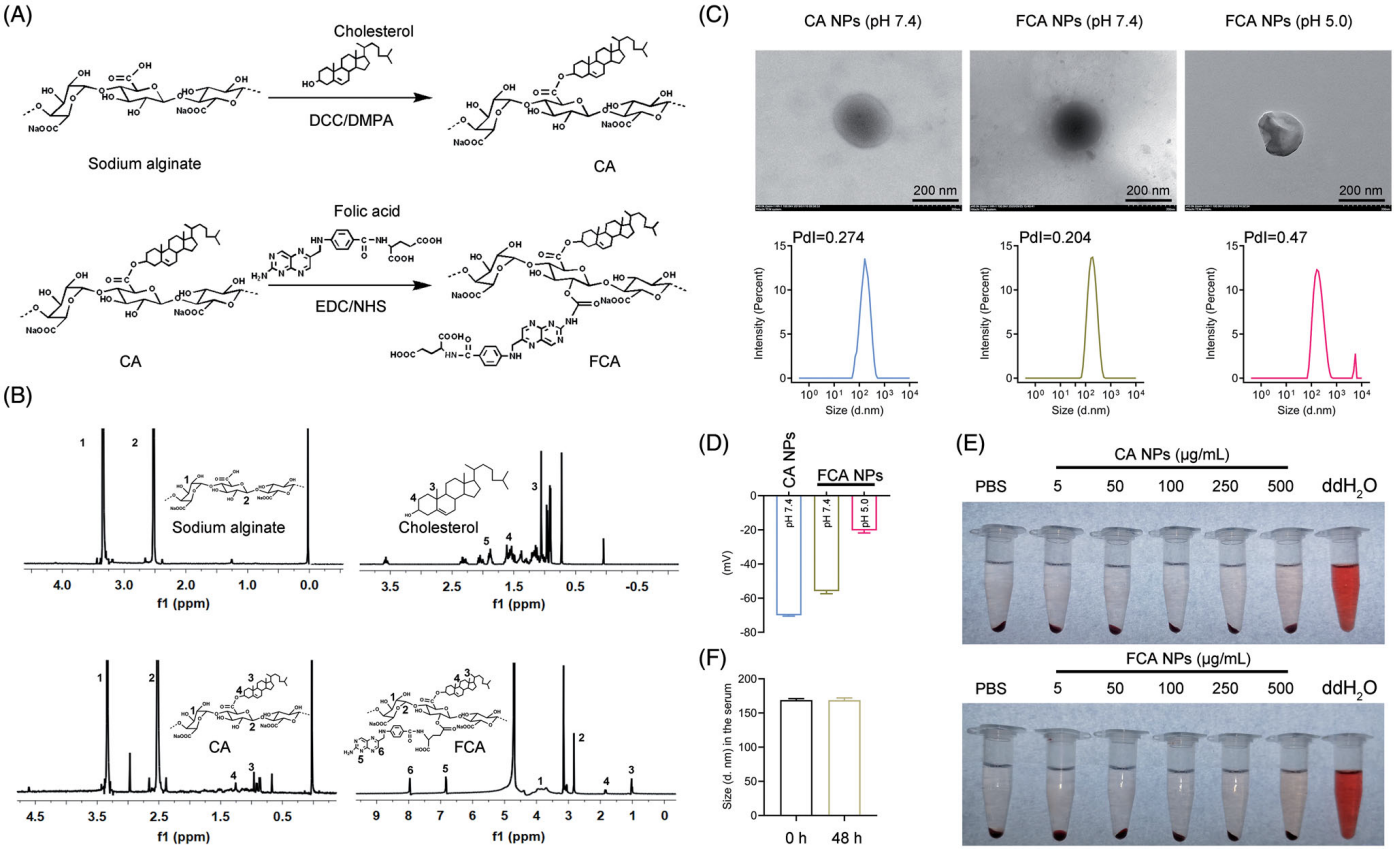
Preparation and characterization of the FCA NPs. (A) Schematic illustration of the formulation of FCA. (B) 1H NMR spectra of FCA. (C) Representative TEM images of the CA NPs, FCA NPs (pH 5.0) and FCA NPs (pH 7.4) (scale bars: 200 nm). Size distributions of the CA NPs, FCA NPs (pH 5.0) and FCA NPs (pH 7.4) measured using a Mastersizer Micro. (D) Zeta-potentials of the CA NPs, FCA NPs (pH 5.0) and FCA NPs (pH 7.4). (E) Intuitive images of the hemolysis assay. (F) Stability of the FCA NPs in the serum at indicated times, *n* = 3 for each group. All values are presented as the mean ± SD.

Hydrophobic cholesterol and hydrophilic folic acid were grafted onto sodium alginate to prepare a melanoma-targeting amphiphilic FCA polymer; a schematic diagram of the preparation procedure for FCA is presented in Figure 1(A). The synthetic procedure for the FCA NPs was divided into two steps: 1) cholesterol-grafted sodium alginate (CA) was synthesized through a reaction between the hydroxyl group of cholesterol and the carboxylic acid group of sodium alginate. 2) Folic acid was conjugated to CA by a carbodiimide-mediated amine coupling reaction. The ^1^H NMR spectrum of FCA shows peaks at 2.5–3.5 ppm corresponding to the hexuronic acid residues of the alginate main chains. The peaks at 0.8–0.9 ppm, 1.2 ppm, and 1.6–1.8 ppm can be attributed to the -CH_3_, -CH_2_, and -CH groups of cholesterol. The peaks observed at 6.6–8.5 ppm indicate the successful conjugation of folic acid (Figure 1(B)). After self-assembly into a core/shell structure in an aqueous solution (pH = 7.4), the hydrodynamic sizes of the CA and FCA NPs were smaller than 180 nm with a narrow size distribution (PDI < 0.3), as demonstrated by the TEM images and Mastersizer analysis (Figure 1(C,D)). The zeta potential of the NPs was −54.3 mV, indicating a strong negatively charged surface, which would reduce the nonspecific binding affinity toward host cells. Notably, the ester and amide bonds could be broken in an acidic environment. The size and zeta potential changes of the FCA NPs were monitored in acidic conditions below pH 5.0. Under these conditions, the average size of the FCA NPs significantly increased from 160 nm to 218 nm. Notably, a FCA NP with a diameter larger than 5000 nm was observed indicating that the nanostructure was impaired in response to the acidic administration environment (Figure 1(C)). The zeta potential remarkably increased to −22 mV at pH 5.0 (Figure 1(D)) because of the hydrolyzation of amide bond and the detachment of folic acid. In addition, to address the biocompatibility of the FCA NPs, a hemolysis test was performed to evaluate the impact of the FCA NPs on the integrity of the RBCs. As shown in Figure 1(E), neither the CA NPs nor FCA NPs exerted any hemolytic properties up to 500 μg/mL.

It should be noted that particles incubate with serum could form a layer of protein corona, leading to increased particle size and reduced targeting ability (Xiao et al., 2018; Xiao & Gao 2018). In our study, we found similar particle sizes of FCA NPs after 48-h incubation in serum (Figure 1(F)), indicating that less protein crown existed on the surface of FCA NPs. This inconsistency may be due to the following potentials, when the protein crown is formed on the surface of NPs. 1. The surface property of NPs is a critical factor for the formation of protein crown. For example, gold NPs interact with proteins through Wan der Waals or π–π interactions, since the hydrophobic properties of their surfaces (Saha et al., 2016). To solve this problem, modification of these NPs by hydrophilic reagents, including the PEG, has been performed and significantly decreases the synthesis of protein crown (Xiao et al., 2018). Similarly, we used sodium alginate, a natural anionic polysaccharide, to provide a hydrophilic shell for FCA NPs, hence, reducing the possibility of the protein crown formation on its surface. 2. A group of positive-charged NPs favors to form protein crown with the serum because of the electrostatic action (Huang et al., 2017). We found that FCA NPs exhibited negatively charged surface, which would also reduce the nonspecific binding toward serum proteins. 3. The fundamental materials are the determinants for the NP properties, as well as the protein crown formation possibilities. Importantly, the carbohydrate-based NPs are reported to hardly adsorb serum proteins to form protein crowns on their surface (Kang et al., 2015). In our study, the basic materials of FCA NPs were all classified as the carbohydrates, thus leading to reduced possibilities to form protein crown. Based on these potentials, we believe that our FCA NPs possess less protein crowns on their surfaces. Meanwhile, similar results were observed in other studies, confirming that the sizes of NPs are stable within the serum (Zheng et al., 2019; York et al., 2012).

Indeed, we could not totally exclude the potentials of protein crown formation and are not able to dissect the detailed sites of protein crowns on NP surface. However, based on the aforementioned reasons, such protein crowns could not completely cover the surface of FCA NPs. Hence, the exposed FA would slave the FCA NPs to the melanoma because of the abundant folate receptors on the membranes of melanoma cells.

Collectively, given the acidic microenvironment around the tumors, these results suggest that our acid-sensitive FCA NPs are stable in the circulation system and are potentially suitable drug carriers for tumor treatments.

### 3.2. *In vitro* and *in vivo* toxicity of the FCA NPs

**Figure 2.**
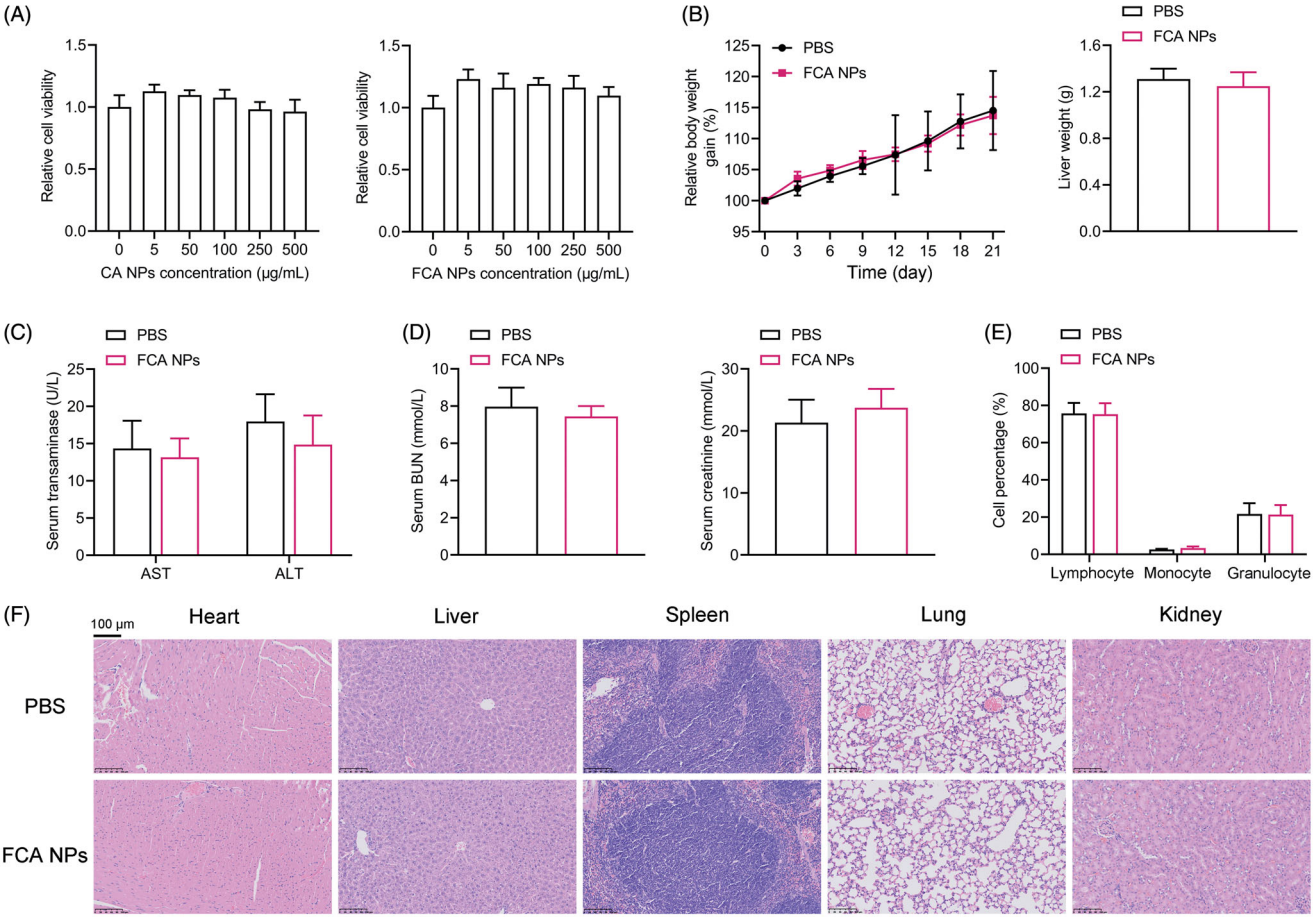
In vitro and in vivo toxicity of the FCA NPs. (A) Cell viability of the human A375 cells treated with indicated doses of CA NPs and FCA NPs. The ND mice were treated with FCA NPs (25 mg/kg body weight, *i.v.* injected every 3 days) for a total of 21 days. (B) Body weight gain. (C) Serum levels of transaminases. (D) Serum levels of creatinine and BUN. (E) CBC analysis. (F) Representative images of the H&E staining for the heart, liver, spleen, lung, and kidney sections (scale bars: 100 μm); *n* = 6 for each group. All values are presented as the mean ± SD.

To exclude the possibility that the FCA NPs have inherent anti-tumor effects, we treated human A375 and SK-MEL-28 melanoma cells with different doses of FCA NPs. As shown in Figure 2(A), neither the CA or FCA NPs show significant cell toxicity at the tested concentrations, suggesting that the FCA NPs modestly affect melanoma cell viability and are safe carriers for further *in vitro* drug delivery tests. To further evaluate the safety *in vivo*, C57BL6/J mice were treated with the FCA NPs (25 mg/kg body weight, intravenously (*i.v.*) injected every 3 days) for a total of 21 days and sacrificed 1 day after the last injection. As shown in Figure 2(B), our FCA NPs modestly affect the body weight of the mice, when compared to that of the PBS-treated control groups. Similarly, we found no serological alterations in the injury-associated parameters, including the liver injury markers—ALTand AST (Figure 2(C)) as well as kidney injury markers—BUN and creatinine (Figure 2(D)). The complete blood count (CBC) results showed no significant difference between the groups, indicating the mild effect of the FCA NPs on the immune system (Figure 2(E), Table S1). Histologically, no significant organic lesions were observed in any of the examined tissues, including the heart, liver, lung, and kidney, of the mice administered either FCA NPs or PBS (Figure 2(F)). These results collectively indicate that the FCA NPs possess satisfactory biosafety and are suitable carriers for drug delivery.

### 3.3. Cellular distribution and uptake of the FCA NPs

**Figure 3.**
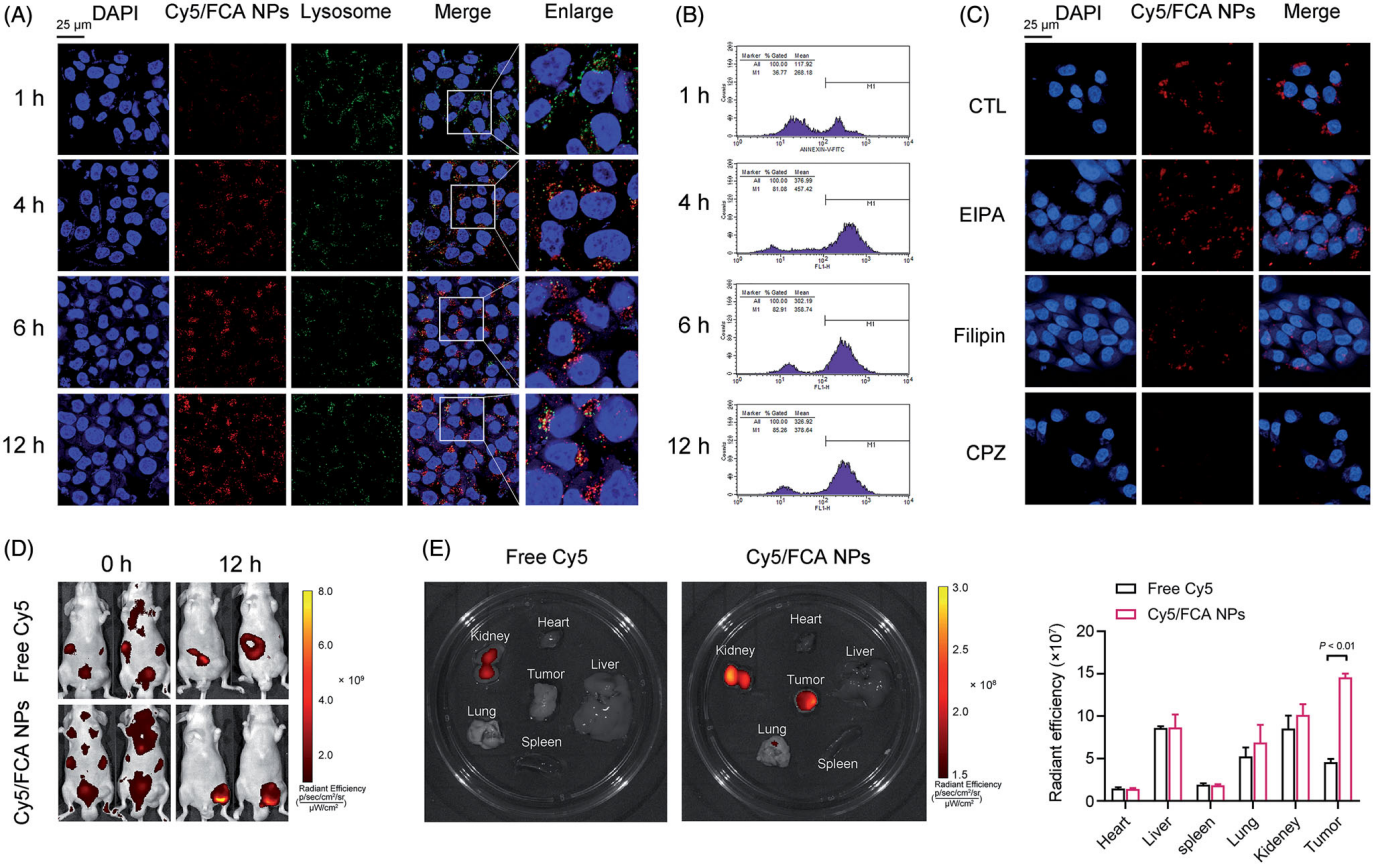
Cellular distribution and uptake of the FCA NPs. (A) CLSM images of the colocalization of human A375 cells and FCA NPs for 1, 4, 6, and 12 h (scale bars: 25 μm). (B, C) Cell uptake and quantitative analysis of the mean fluorescence intensity by flow cytometry for 1, 4, 6, and 12 h. (D) Representative fluorescence microscopy images of human A375 cells treated with Cy5-labeled FCA NPs exclusively or in combination with indicated inhibitors (scale bars: 25 μm). (E) Fluorescence imaging of the biodistribution of the FCA NPs in vivo. (F) Fluorescence imaging of the major organs and tumors. Cy5-labeled FCA NPs: Cy5/FCA NPs.

The cellular distribution and uptake of nanocarriers reflect their ability to deliver drugs intracellularly. To determine these characteristics, the FCA NPs were labeled with Cy5 to study their cellular distribution in A375 melanoma cells. As shown in Figure 3(A), the red fluorescence signal of Cy5 was weak in the cells after 1 h of FCA NP incubation, while the colocalization between the FCA NPs and cells became more apparent over time. After 12 h of treatment, the red fluorescence signals significantly increased, suggesting that more drugs could be delivered into the cells. Meanwhile, the Cy5-labeled FCA NPs were colocalized with Lyso-Tracker (a lysosomal probe) and turned yellow, suggesting that the NPs were captured by lysosomes in the acidic environment, resulting in the release of the drug. Furthermore, the cellular uptake efficiency of the Cy5-labeled FCA NPs was measured using flow cytometry analysis. Similarly, the fluorescence intensity of the FCA NPs in the A375 cells increased with prolonged incubation times (Figure 3(B)). Notably, extracellular cargo is internalized by mammalian cells through endocytosis and macropinocytosis. To elucidate the cellular uptake mechanisms of the FCA NPs, A375 cells were incubated with Cy5-labeled FCA NPs in the presence of specific inhibitors of the endocytotic processes. As shown in Figure 3(C)), the cellular uptake of the FCA NPs was almost abolished by chlorpromazine, an inhibitor of clathrin-mediated endocytosis. In addition, other inhibitors, such as filipin (a caveolae-mediated endocytosis inhibitor) and EIPA (a macropinocytosis inhibitor), slightly altered the cellular uptake of the FCA NPs. These results suggest that clathrin-mediated endocytosis is an essential and dominant process in the cellular uptake of FCA NPs.

### 3.4. Tumor targeting specificity and efficiency of the FCA NPs *in vivo*

To estimate the tumor-targeting specificity of the FCA NPs *in vivo*, tumor-bearing nude mice were injected with either Cy5-labeled FCA NPs (250 μg/mL) or an equivalent of Cy5 for 12 h. The detection time was chosen according to previous studies, which indicated that circulating folic acid was functionally accumulated in the xenograft. As shown in Figure 3(D), the fluorescence signals of free Cy5 are widely accumulated in the liver, spleen, and kidney. In contrast, the fluorescence of the Cy5-labeled FCA NPs was condensed in the xenograft melanoma tumors. Moreover, *ex vivo* imaging analysis of the excised tumors and organs revealed that the fluorescence signals of Cy5-labeled FCA NPs were more intense in the tumors (Figure 3(E)), suggesting remarkable tumor-targeting specificity. In addition, these fluorescence signals were observed in the livers and kidneys of the mice (Figure 3(E)), suggesting that the FCA NPs are primarily captured by the liver and excreted by renal tissues.

### 3.5. Drug loading and release

The drug loading capacity of the FCA NPs was examined to evaluate their potential as drug carriers. As shown in Figure S1A,B, the MET loading efficiency of the FCA NPs was upregulated with an increase in the MET concentration from 5 to 20 mg/mL and was stabilized when the MET concentration was over 20 mg/mL. Notably, when the mass ratio of FCA to MET was 1:4, the optimal MET LE and drug EE was 3.3% and approximately 82.8%, respectively. Additionally, the LE of DOX was 14.0%, whereas that of DOX in the FCA NPs was 69.5%. Furthermore, to evaluate the performance of the FCA NP-controlled drug release, the concentrations of MET and DOX released were analyzed using dialysis. Drug-containing FCA NPs were dispersed in two buffer solutions, pH 5.0 and pH 7.4. The cumulative release of MET and DOX significantly increased in the pH 5.0 solution after 240 min of incubation. In brief, 75.8% of DOX was released in the pH 5.0 solution, whereas only 40.4% of DOX was released from the FCA NPs when the solution pH was 7.4 (Figure S1C). Surprisingly, the release of MET was only approximately 7.0% in the pH 7.4 solution. Conversely, when the pH of the solution was 5.0, MET was rapidly released from the FCA NPs with a release efficiency of 32.50% (Figure S1D). These results further confirm that the FCA NPs are pH-sensitive nanocarriers that stably exist in the circulation system. Functionally, they can rapidly release MET and DOX in an acidic microenvironment to suppress tumor growth. Furthermore, in order to quantify the kinetics of the MET and DOX release from FCA NPs, zero-order, first-order, and Higuchi models were fitted to the drug release curve. As shown in Table S2, the results show that the function calculated using the drug release doses and times mostly fitted the highest linearity, which resulted in the highest linearity and first-order kinetics model the best fitting effect.

### 3.6. Evaluation of the antitumor activity *in vitro*

**Figure 4.**
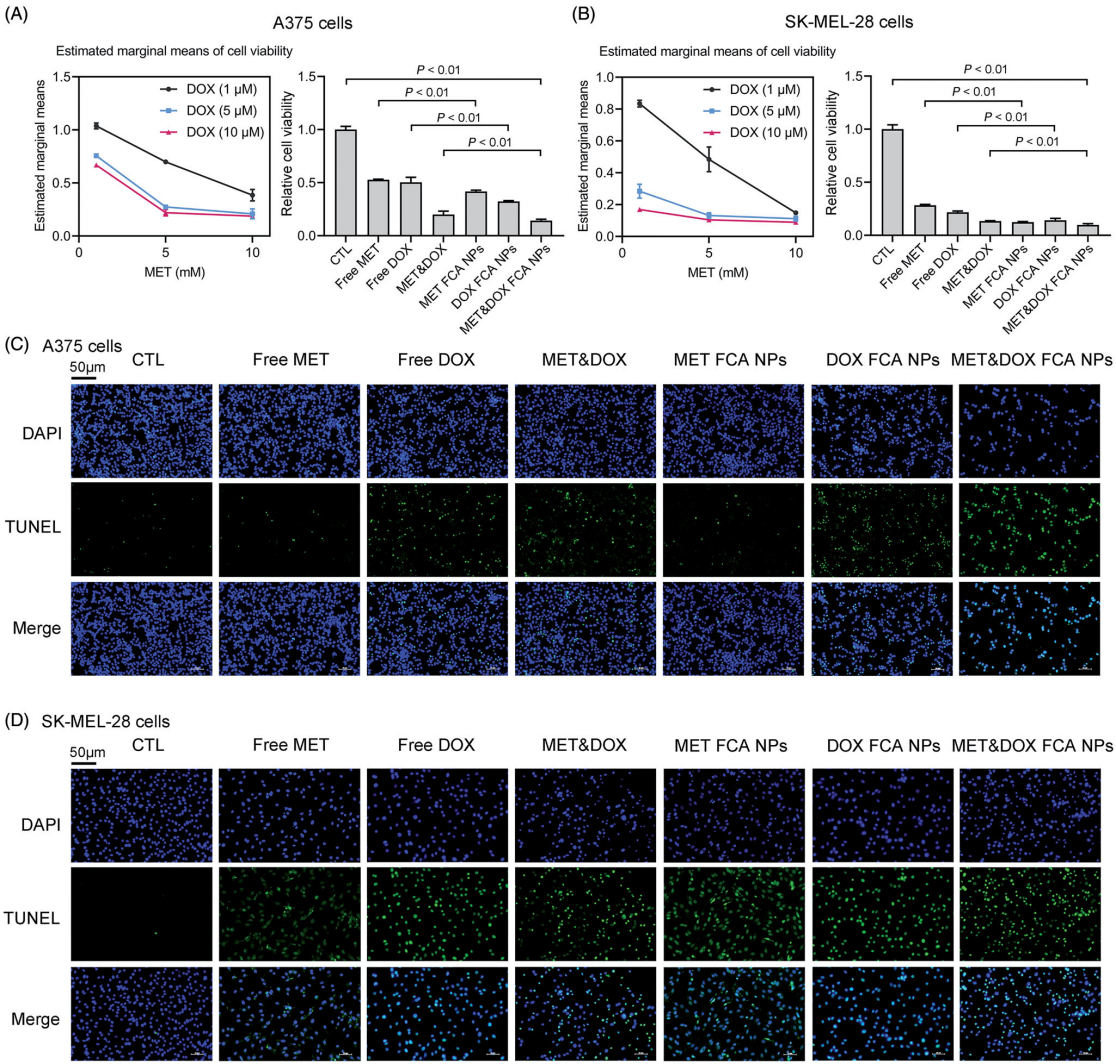
Evaluation of the antitumor activity in vitro. (A) Cell viability of the human A375 cells treated with free MET (*x*-axis). The viability of the A375 cells treated with MET, DOX, MET&DOX, FCA NP-loaded MET, FCA NP-loaded DOX, and FCA NP-loaded MET&DOX. (B) Cell viability of the human SK-MEL-28 cells treated with free MET (*x*-axis) and free DOX. The viability of the SK-MEL-28 cells treated with MET, DOX, MET&DOX, FCA NP-loaded MET, FCA NP-loaded DOX, and FCA NP-loaded MET&DOX. All values are presented as the mean ± SD. (C, D) TUNEL staining analysis of the human A375 and SK-MEL-28 cells after treatment with MET, DOX, MET&DOX, FCA NP-loaded MET, FCA NP-loaded DOX, and FCA NP-loaded MET&DOX. Scale bars: 50 μm. *n* = 6 for each group. CTL: FCA NPs, MET FCA NPs: FCA NP-loaded MET, DOX FCA NPs: FCA NP-loaded DOX, MET&DOX FCA NPs: FCA NP-loaded MET&DOX.

To examine the effects of the FCA NP-loaded MET&DOX on melanoma progression, we treated A375 and SK-MEL-28 cells with either free MET (10 mM) and DOX (10 μM), or a combination of the two, loaded in the FCA NPs or as free drugs. As shown in Figure 4(A,B), CCK-8 analysis indicated that the combination of MET&DOX (MET: 5 mM, DOX: 5 μM) synergistically induced a remarkable reduction in the A375 and SK-MEL-28 cell viability. In addition, such a synergistic effect was consolidated by half-maximal inhibitory concentration (IC_50_) analysis using a well-established calculation model described by Yang et al. (Yang et al., 2019) (Figure S2). Moreover, the FCA NPs single-loaded with MET or DOX exhibited antitumor effects comparable to those of the free MET&DOX combination-treated group; this suggests that our FCA NPs increased the anti-tumor properties of the drugs. Notably, treatment with FCA NP-loaded MET&DOX caused a greater inhibition of the A375 and SK-MEL-28 cell viabilities, when compared to the free MET&DOX treatment (Figure S2, Table S3-5). Similar results were observed in the TUNEL assay (Figure 4(C,D)). These results suggest that FCA NPs increased the anti-tumor effects of the MET&DOX combination *in vitro*.

### 3.7. Evaluation of the antitumor activity *in vivo*

**Figure 5.**
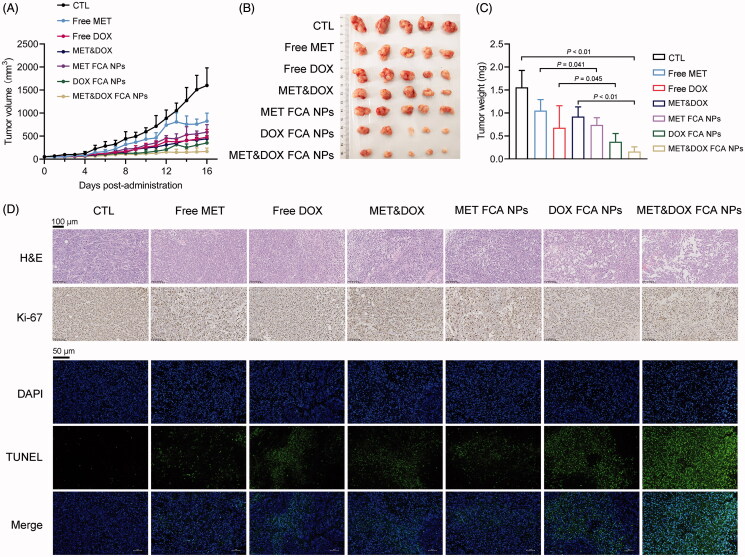
Evaluation of the antitumor activity *in vivo*. A xenograft melanoma tumor model was established. Different drug formulations were *i.v.* injected into the mice at 2-day intervals over 16 days. (A) Tumors volumes during the treatment. (B) Photos of tumor-bearing mice and dissected tumors after the16-day treatment. (C) Tumor weight. (D) H&E, Ki-67 (scale bars: 100 μm) and TUNEL (scale bars: 50 μm) staining of the corresponding tumor tissues obtained after different treatments. *n* = 5 for each group. CTL: FCA NPs, MET FCA NPs: FCA NP-loaded MET, DOX FCA NPs: FCA NP-loaded DOX, MET&DOX FCA NPs: FCA NP-loaded MET&DOX.

Next, we investigated the antitumor therapeutic efficacy of FCA NP-loaded MET&DOX *in vivo*. A unilateral melanoma tumor model was established and divided into seven groups, as in the *in vitro* experiment. Different drug formulations were *i.v.* injected into the mice eight times with 2-day intervals, and the tumor volumes monitored before each injection. None of the drug treatments altered the body weight of the mice or hepatic AST and ALT levels during the experimental period. (Figure S3A,B). Additionally, no significant pathological abnormalities were observed in the peripheral tissues, including the heart, liver, spleen, lung, and kidney (Figure S3C). These results suggest that the FCA NP-loaded MET&DOX combination is biologically safe *in vivo*. As shown in Figure 5(A,B), the tumor volumes of the free MET- and DOX-treated groups decreased by 48.37% and 42.17%, respectively, whereas the combination treatment of free MET and DOX showed a synergistic inhibition effect on the xenograft volumes. Accordingly, FCA NP-loaded MET or DOX exhibited comparable effects to the free MET&DOX combination-treated group. Coinciding with the *in vitro* results, the FCA NP-loaded MET&DOX combination exerted the strongest tumor-inhibitory properties on the tumor volume (89.54% inhibition compared to the control group) among all the drug-treated groups. Consistently, the xenograft tumor weights were reduced by 89.56% (Figure 5(C), Table S6). Histologically, H&E staining analysis showed that the tumor cell density and blurred tumor cell borders dramatically decreased in all the drug-treated groups, while the FCA NP-loaded MET&DOX combination showed the strongest effects among all groups. IHC and TUNEL analyses further confirmed that the FCA NPs increased the anti-tumor properties of MET and DOX as well as their combination (Figure 5(D)).

### 3.8. Fca NP-loaded MET&DOX induces melanoma cell death via induction of pyroptosis, apoptosis, and necroptosis (PANoptosis)

**Figure 6.**
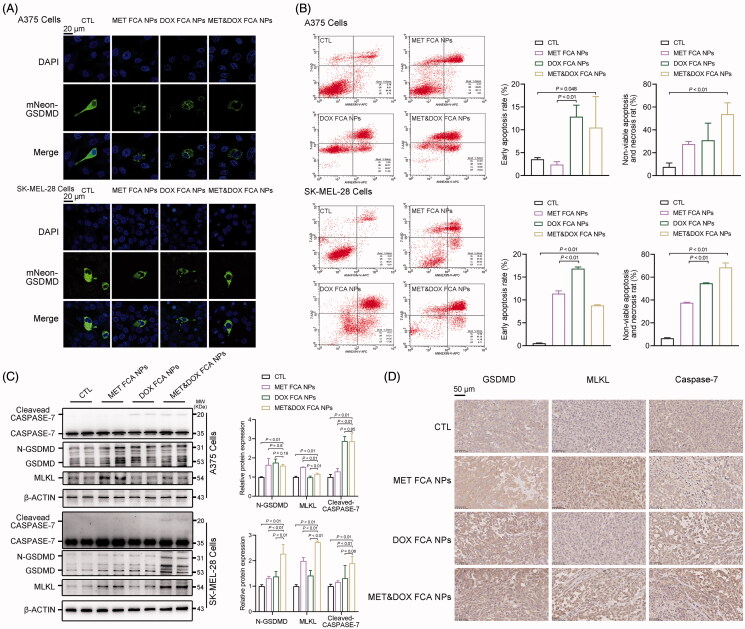
FCA NP-loaded MET&DOX induces melanoma cell death via induction of pyroptosis, apoptosis, and necroptosis (PANoptosis). (A) Representative confocal microscopy images of cleaving mNeon-GSDMD expressed in human A375 and SK-MEI-28 cells, scale bars: 20 μm. (B) Apoptosis and necroptosis of human A375 and SK-MEL-28 cells following 24-h incubation with different formulations; *n* = 3 for each group. (C) The protein expression levels of the GSDMD, CASPASE-7, and MLKL protein in human A375 and SK-MEL-28 cells following 24 h incubation with different formulations; *n* = 4 for each group. (D) IHC analysis of the GSDMD, Caspase-7, and MLKL protein expression in the corresponding tumor tissues obtained after the different treatments; scale bars: 50 μm, *n* = 5 for each group. CTL: FCA NPs, MET FCA NPs: FCA NP-loaded MET, DOX FCA NPs: FCA NP-loaded DOX, MET&DOX FCA NPs: FCA NP-loaded MET&DOX.

To further investigate the types of FCA NP-loaded MET&DOX-triggered melanoma cell death, we evaluated the three main types of cell death, pyroptosis, apoptosis, and necroptosis (PANoptosis) (Karki et al., 2020). As shown in Figure 6(A), either FCA NP-loaded MET, FCA NP-loaded DOX, or their combination increased the positive fluorescence signals of the N-GSDMD effector domain. Flow cytometry analysis revealed that FCA NP-loaded MET triggered both non-viable apoptosis and necrosis in the melanoma cells, whereas FCA NP-loaded DOX initiated cell apoptosis at an early stage (68.37% for A375 cells and 67.60% for SK-MEL-28 cells) (Figure 6(B), Table S7-10). As expected, the FCA NP-loaded MET&DOX combination consistently exhibited the strongest effects among all the groups. At the molecular level, MET increased the protein expression of N-GSDMD and MLKL, while DOX induced significant protein expression of N-GSDMD and active CASPASE-7. Meanwhile, the combination of FCA NP-loaded MET&DOX triggered the activation of PANoptosis-associated proteins (Figure 6(C)). Similar PANoptosis results for MET and DOX were achieved in the xenograft melanoma tumors (Figure 6(D)), further supporting the hypothesis that FCA NP-loaded MET&DOX triggers PANoptosis in melanoma cells.

## 4. Conclusion

In this study, a novel nanodrug carrier based on a new degradable and biocompatible homopolymer, FCA, was established, and its biosafety and biodistribution were evaluated. Taking advantage of the nanosystem, we successfully co-delivered MET&DOX into xenograft melanoma tumors and increased their anti-melanoma effects. Mechanistically, we found that FCA NP-loaded MET&DOX triggered PANoptosis of melanoma cells *in vitro* and *in vivo*. Our findings provide a state-of-the-art melanoma tumor-specific nanosystem that co-delivers MET&DOX combinations for melanoma treatment.

## Supplementary Material

Supplemental Material
