# Supplementary material for: Self-assembled polymeric nanocarrier-mediated co-delivery of metformin and doxorubicin for melanoma therapy
Source: Drug Deliv. 2021 Mar 17;28(1):594–606. doi: 10.1080/10717544.2021.1898703 (PMC7996084; doi:10.1080/10717544.2021.1898703)
Supplement: Supplemental Material [file IDRD_A_1898703_SM7509.docx]

**Supporting information**

**Self-assembled polymeric nanocarrier-mediated co-delivery of** **metformin and doxorubicin for** **melanoma therapy**

Mingming Song, Wentao Xia, Zixuan Tao, Bin Zhu, Wenxiang Zhang, Chang Liu*, Siyu Chen*

**
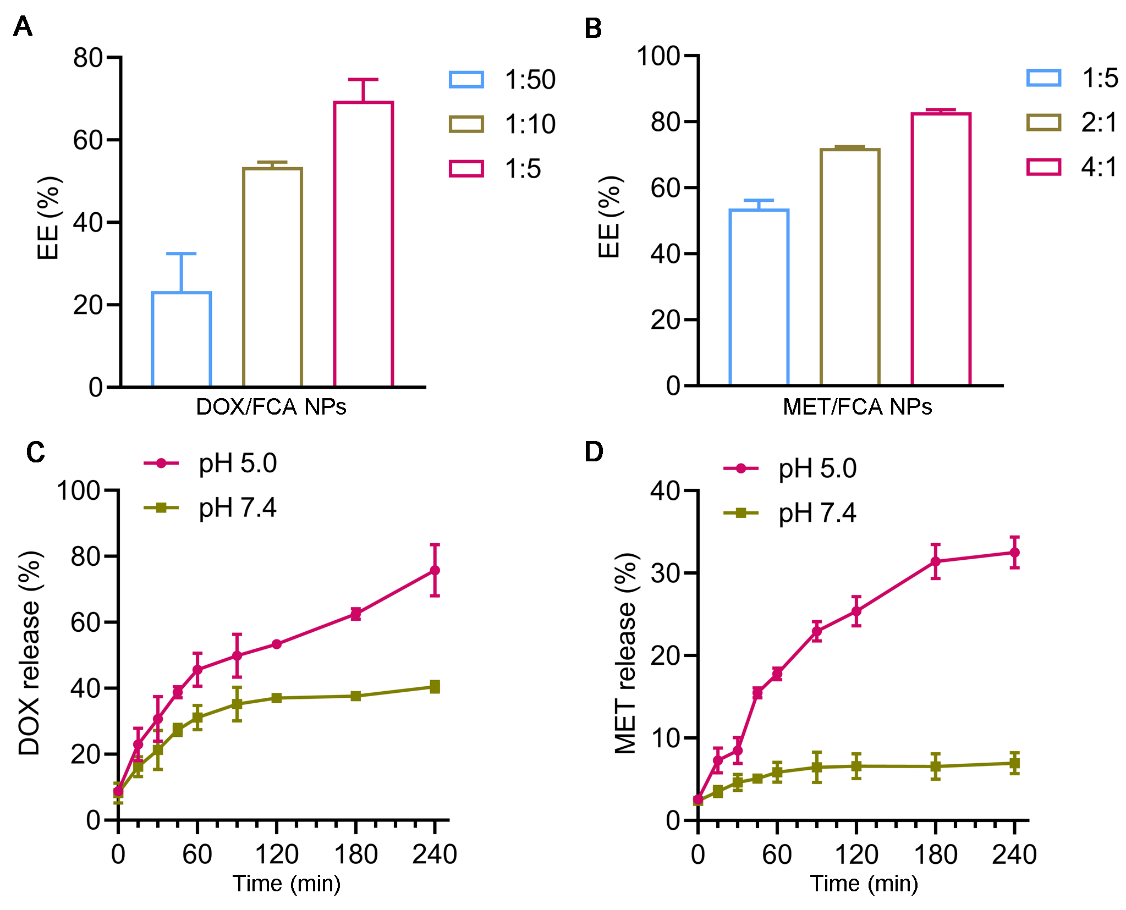
**

**Figure S1.** Drug loading and release. A, B) EE% and LE%. C) *In vitro* DOX release from the FCA NPs in PBS at different pH values. D) *In vitro* MET release from the FCA NPs in PBS at different pH values. All values are presented as the mean ± SD.


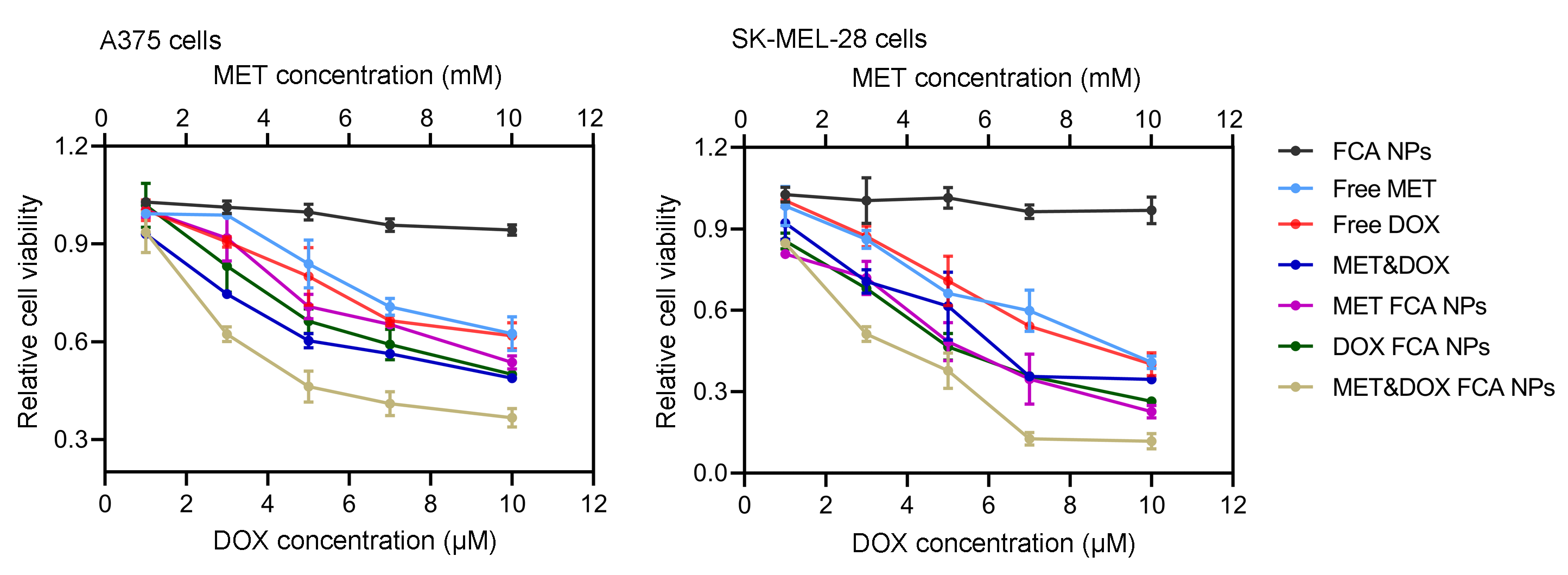


**Figure S2.** Synergistic therapeutic efficacy of the MET&DOX FCA NPs *in vitro*. All values are presented as the mean ± SD.


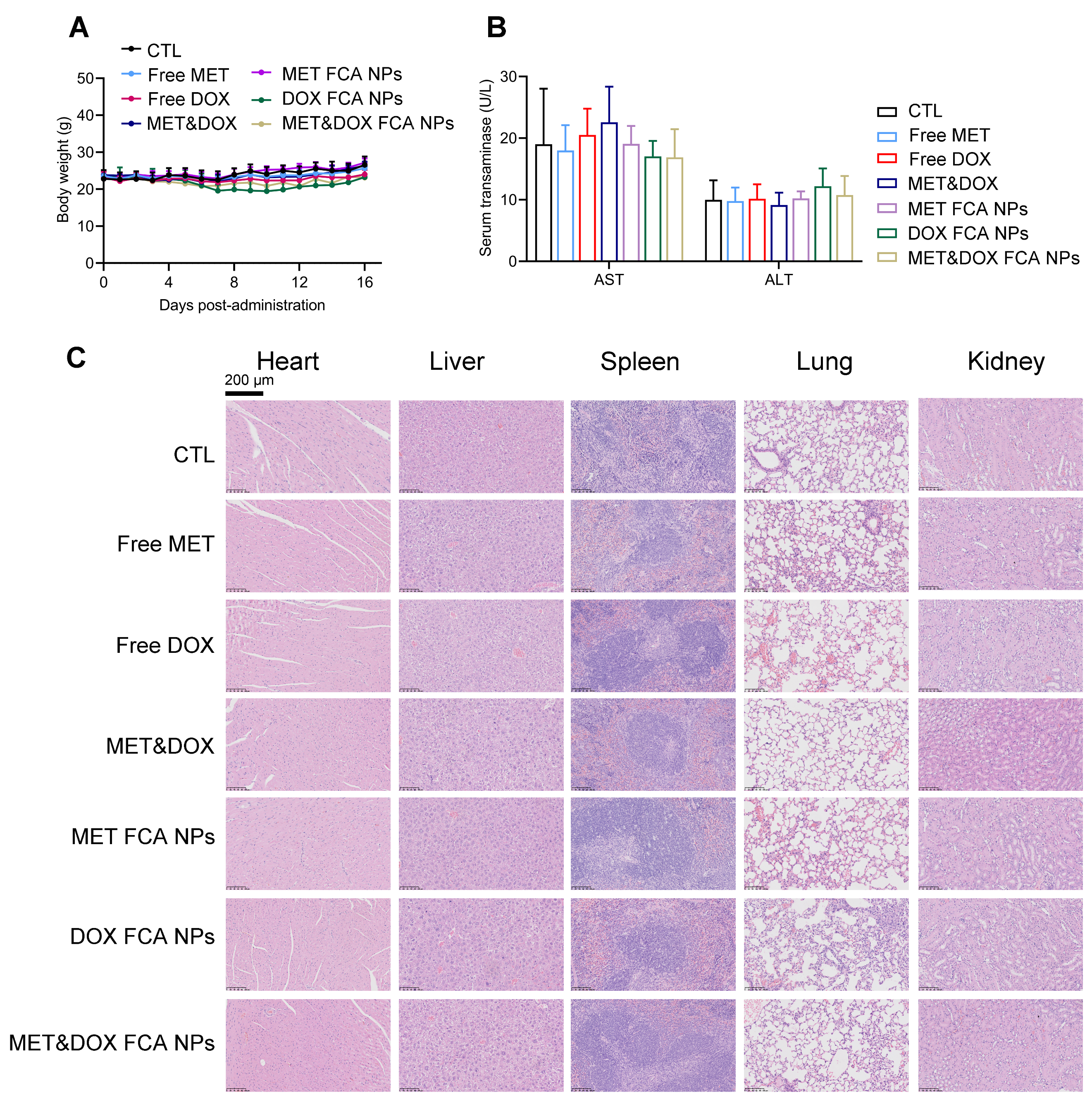


**Figure S3.** A xenograft melanoma tumor model was established. Different drug formulations were i.v. injected into the mice at 2-day intervals over 16 days. A) Mouse body weight. B) Serum transaminases. C) H&E analysis of mouse heart, liver, spleen, lung and kidney, scale bars: 200 μm, n=5 for each group. CTL: NPs, MET NPs: FCA NP-loaded MET, DOX NPs: FCA NP-loaded DOX, MET&DOX NPs: FCA NP-loaded MET&DOX

**Table S1.** CBC analysis.

| Name | PBS-1 | PBS-2 | PBS-3 | PBS-4 | PBS-5 | PBS-6 | FCA NPs-1 | FCA NPs-2 | FCA NPs-3 | FCA NPs-4 | FCA NPs-5 | FCA NPs-6 | Units | CI |
| --- | --- | --- | --- | --- | --- | --- | --- | --- | --- | --- | --- | --- | --- | --- |
| WBC | 6.8 | 8.1 | 9.6 | 6.7 | 4.5 | 6.1 | 5.8 | 5 | 6 | 7.7 | 7.5 | 6.7 | 10^9/L | 0.8-6.8 |
| Lymph# | 5.2 | 5.8 | 7 | 6.1 | 3.3 | 4.6 | 3.9 | 3.6 | 4.7 | 5.6 | 5.7 | 5.7 | 10^9/L | 0.7-5.7 |
| Mon# | 0.1 | 0.2 | 0.2 | 0.9 | 0.1 | 0.1 | 0.3 | 0.2 | 0.2 | 0.3 | 0.3 | 0.1 | 10^9/L | 0.0-0.3 |
| Gran# | 1.5 | 2.1 | 2.4 | 3 | 1.1 | 1.4 | 1.6 | 1.2 | 1.1 | 1.8 | 1.5 | 0.9 | 10^9/L | 0.1-1.8 |
| Lymph% | 75.9 | 71.3 | 72.8 | 86.8 | 73.3 | 74 | 67.3 | 72.4 | 78.3 | 72.9 | 76.4 | 84.6 | % | 55.8-90.6 |
| Mon% | 2.4 | 3 | 2.6 | 3.1 | 2.4 | 2.4 | 4.8 | 3.5 | 2.7 | 3.3 | 3.5 | 2.2 | % | 1.8-6.0 |
| Gran% | 21.7 | 25.7 | 24.6 | 10.1 | 24.3 | 23.6 | 27.9 | 24.1 | 19 | 23.8 | 20.1 | 13.2 | % | 8.6-38.9 |
| RBC | 8.65 | 7.7 | 8.3 | 8.51 | 7.2 | 8.16 | 6.67 | 5.5 | 6.13 | 7.77 | 7.75 | 8.09 | 10^12/L | 6.36-9.42 |
| HGB | 137 | 127 | 136 | 134 | 117 | 131 | 108 | 88 | 101 | 127 | 125 | 129 | g/L | 110-143 |
| HCT | 43.5 | 39.1 | 42 | 42.8 | 36 | 41.5 | 32.5 | 26.6 | 30 | 39 | 39.4 | 40.9 | % | 34.6-44.6 |
| MCV | 50.4 | 50.9 | 50.7 | 50.3 | 50 | 50.9 | 48.8 | 48.5 | 49.1 | 50.2 | 50.9 | 50.6 | fL | 48.2-58.3 |
| MCH | 15.8 | 16.4 | 16.3 | 15.7 | 16.2 | 16 | 16.1 | 16 | 16.4 | 16.3 | 16.1 | 15.9 | pg | 15.8-19 |
| MCHC | 314 | 324 | 323 | 313 | 325 | 315 | 332 | 330 | 336 | 325 | 317 | 315 | g/L | 302-353 |
| RDW | 15.1 | 13.6 | 14.9 | 14.5 | 15.1 | 14.5 | 12.8 | 12.8 | 13.5 | 14.2 | 13.6 | 13.9 | % | 13-17 |
| PLT | 964 | 823 | 801 | 1017 | 575 | 623 | 560 | 378 | 347 | 524 | 730 | 594 | 10^9/L | 450-1590 |
| MPV | 5.8 | 5.7 | 5.8 | 5.7 | 6.1 | 5.8 | 5.8 | 5.8 | 5.9 | 6 | 5.6 | 5.7 | fL | 3.8-6.0 |
| PDW | 17.4 | 17.5 | 17.3 | 17.4 | 17.5 | 17.4 | 17.6 | 17.6 | 17.5 | 17.7 | 17.6 | 17.6 |  |  |
| PCT | 0.559 | 0.469 | 0.464 | 0.579 | 0.35 | 0.361 | 0.324 | 0.219 | 0.204 | 0.314 | 0.408 | 0.338 | % |  |

**Supplementary Table 2.** Drug release kinetics characteristic of FCA NPs.

| DOX release kinetics characteristic of FCA NPs | | | |
| --- | --- | --- | --- |
| pH | Model | Equation | R^2^ |
| 5 | Zero order kinetics | R%=0.2407t+22.87 | 0.8896 |
|  | First order kinetics | Ln(1-R%)=-0.00049t+4.3392 | 0.9648 |
|  | Higuchi equation | R%=4.2338t^1/2^+8.7347 | 0.9879 |
| 7.4 | Zero order kinetics | R% = 0.1165t + 18.184 | 0.7248 |
|  | First order kinetics | Ln(1-R%) = -0.0016t + 4.4028 | 0.7661 |
|  | Higuchi equation | R% = 2.1919t^1/2^ + 10.461 | 0.9215 |
| MET release kinetics characteristic of FCA NPs | | | |
| pH | Model | Equation | R^2^ |
| 5 | Zero order kinetics | R% = 0.1257t + 7.3037 | 0.8887 |
|  | First order kinetics | Ln(1-R%) = -0.0016t + 4.5328 | 0.915 |
|  | Higuchi equation | R%=2.1861t^1/2^ + 0.4318 | 0.9642 |
| 7.4 | Zero order kinetics | R% = 0.0161t + 3.9329 | 0.6764 |
|  | First order kinetics | Ln(1-R%) = -0.0002t + 4.565 | 0.6808 |
|  | Higuchi equation | R% = 0.3089t^1/2^ + 2.8178 | 0.893 |

**Supplementary Table 3.** Ordinary one-way ANOVA Multiple comparisons (A375 cell viability).

| Two-stage linear step-up procedure of Benjamini, Krieger and Yekutieli | Mean Diff. | Discovery? | q value | Individual *P* Value |
| --- | --- | --- | --- | --- |
| CTL vs. Free MET | 0.4731 | Yes | <0.0001 | <0.0001 |
| CTL vs. Free DOX | 0.4966 | Yes | <0.0001 | <0.0001 |
| CTL vs. MET&DOX | 0.8002 | Yes | <0.0001 | <0.0001 |
| CTL vs. MET FCA NPs | 0.5831 | Yes | <0.0001 | <0.0001 |
| CTL vs. DOX FCA NPs | 0.6775 | Yes | <0.0001 | <0.0001 |
| CTL vs. MET&DOX FCA NPs | 0.8586 | Yes | <0.0001 | <0.0001 |
| Free MET vs. Free DOX | 0.0235 | Yes | 0.0064 | 0.1271 |
| Free MET vs. MET&DOX | 0.3271 | Yes | <0.0001 | <0.0001 |
| Free MET vs. MET FCA NPs | 0.11 | Yes | <0.0001 | <0.0001 |
| Free MET vs. DOX FCA NPs | 0.2043 | Yes | <0.0001 | <0.0001 |
| Free MET vs. MET&DOX FCA NPs | 0.3855 | Yes | <0.0001 | <0.0001 |
| Free DOX vs. MET&DOX | 0.3036 | Yes | <0.0001 | <0.0001 |
| Free DOX vs. MET FCA NPs | 0.08647 | Yes | <0.0001 | <0.0001 |
| Free DOX vs. DOX FCA NPs | 0.1808 | Yes | <0.0001 | <0.0001 |
| Free DOX vs. MET&DOX FCA NPs | 0.362 | Yes | <0.0001 | <0.0001 |
| MET&DOX vs. MET FCA NPs | -0.2172 | Yes | <0.0001 | <0.0001 |
| MET&DOX vs. DOX FCA NPs | -0.1228 | Yes | <0.0001 | <0.0001 |
| MET&DOX vs. MET&DOX FCA NPs | 0.05838 | Yes | <0.0001 | 0.0004 |
| MET NPs vs. DOX FCA NPs | 0.09438 | Yes | <0.0001 | <0.0001 |
| MET NPs vs. MET&DOX FCA NPs | 0.2755 | Yes | <0.0001 | <0.0001 |
| DOX FCA NPs vs. MET&DOX FCA NPs | 0.1811 | Yes | <0.0001 | <0.0001 |

**Supplementary Table 4.** Ordinary one-way ANOVA Multiple comparisons (SK-MEL-28 cell viability).

| Two-stage linear step-up procedure of Benjamini, Krieger and Yekutieli | Mean Diff. | Discovery? | q value | Individual *P* Value |
| --- | --- | --- | --- | --- |
| CTL vs. Free MET | 0.7164 | Yes | <0.0001 | <0.0001 |
| CTL vs. Free DOX | 0.7832 | Yes | <0.0001 | <0.0001 |
| CTL vs. MET&DOX | 0.8662 | Yes | <0.0001 | <0.0001 |
| CTL vs. MET FCA NPs | 0.876 | Yes | <0.0001 | <0.0001 |
| CTL vs. DOX FCA NPs | 0.8579 | Yes | <0.0001 | <0.0001 |
| CTL vs. MET&DOX FCA NPs | 0.9025 | Yes | <0.0001 | <0.0001 |
| Free MET vs. Free DOX | 0.06679 | Yes | <0.0001 | <0.0001 |
| Free MET vs. MET&DOX | 0.1498 | Yes | <0.0001 | <0.0001 |
| Free MET vs. MET FCA NPs | 0.1595 | Yes | <0.0001 | <0.0001 |
| Free MET vs. DOX FCA NPs | 0.1415 | Yes | <0.0001 | <0.0001 |
| Free MET vs. MET&DOX FCA NPs | 0.186 | Yes | <0.0001 | <0.0001 |
| Free DOX vs. MET&DOX | 0.083 | Yes | <0.0001 | <0.0001 |
| Free DOX vs. MET FCA NPs | 0.09273 | Yes | <0.0001 | <0.0001 |
| Free DOX vs. DOX FCA NPs | 0.07472 | Yes | <0.0001 | <0.0001 |
| Free DOX vs. MET&DOX FCA NPs | 0.1193 | Yes | <0.0001 | <0.0001 |
| MET&DOX vs. MET FCA NPs | 0.009728 | No | 0.0611 | 0.388 |
| MET&DOX vs. DOX FCA NPs | -0.008282 | No | 0.0693 | 0.4617 |
| MET&DOX vs. MET&DOX FCA NPs | 0.03625 | Yes | 0.0005 | 0.0025 |
| MET NPs vs. DOX FCA NPs | -0.01801 | Yes | 0.019 | 0.1145 |
| MET NPs vs. MET&DOX FCA NPs | 0.02652 | Yes | 0.004 | 0.0227 |
| DOX FCA NPs vs. MET&DOX FCA NPs | 0.04453 | Yes | <0.0001 | 0.0003 |

**Supplementary Table S5.** IC_50_ values analysis.

| Cells | Groups | MET IC_50_ (μM) | DOX IC_50_ (mM) |
| --- | --- | --- | --- |
| A375 Cells | FCA NPs | N/A | N/A |
|  | Free MET | 5.486 | N/A |
|  | Free DOX | N/A | 5.102 |
|  | MET&DOX | 3.542 | 3.542 |
|  | MET FCA NPs | 4.739 | N/A |
|  | DOX FCA NPs | N/A | 4.197 |
|  | MET&DOX FCA NPs | 2.624 | 2.624 |
|  |  |  |  |
| SK-MEL-28 Cells | FCA NPs | N/A | N/A |
|  | Free MET | 10.92 | N/A |
|  | Free DOX | N/A | 6.366 |
|  | MET&DOX | 4.833 | 4.833 |
|  | MET FCA NPs | 5.054 | N/A |
|  | DOX FCA NPs | N/A | 4.317 |
|  | MET&DOX FCA NPs | 3.774 | 3.774 |

**Supplementary Table 6.** Ordinary one-way ANOVA Multiple comparisons (Tumor weight).

| Two-stage linear step-up procedure of Benjamini, Krieger and Yekutieli | Mean Diff. | Discovery? | q value | Individual *P* Value |
| --- | --- | --- | --- | --- |
| CTL vs. Free MET | 0.505 | Yes | 0.0058 | 0.0073 |
| CTL vs. Free DOX | 0.8778 | Yes | <0.0001 | <0.0001 |
| CTL vs. MET&DOX | 0.6338 | Yes | 0.0013 | 0.0011 |
| CTL vs. MET FCA NPs | 0.8154 | Yes | 0.0001 | <0.0001 |
| CTL vs. DOX FCA NPs | 1.183 | Yes | <0.0001 | <0.0001 |
| CTL vs. MET&DOX FCA NPs | 1.396 | Yes | <0.0001 | <0.0001 |
| Free MET vs. Free DOX | 0.3728 | Yes | 0.03 | 0.0416 |
| Free MET vs. MET&DOX | 0.1288 | No | 0.2205 | 0.4667 |
| Free MET vs. MET FCA NPs | 0.3104 | No | 0.0542 | 0.0862 |
| Free MET vs. DOX FCA NPs | 0.6776 | Yes | 0.0008 | 0.0006 |
| Free MET vs. MET&DOX FCA NPs | 0.891 | Yes | <0.0001 | <0.0001 |
| Free DOX vs. MET&DOX | -0.244 | No | 0.0962 | 0.1731 |
| Free DOX vs. MET FCA NPs | -0.0624 | No | 0.3255 | 0.7234 |
| Free DOX vs. DOX FCA NPs | 0.3048 | No | 0.0542 | 0.0917 |
| Free DOX vs. MET&DOX FCA NPs | 0.5182 | Yes | 0.0052 | 0.0061 |
| MET&DOX vs. MET FCA NPs | 0.1816 | No | 0.1527 | 0.307 |
| MET&DOX vs. DOX FCA NPs | 0.5488 | Yes | 0.0037 | 0.0039 |
| MET&DOX vs. MET&DOX FCA NPs | 0.7622 | Yes | 0.0002 | 0.0002 |
| MET NPs vs. DOX FCA NPs | 0.3672 | Yes | 0.03 | 0.0445 |
| MET NPs vs. MET&DOX FCA NPs | 0.5806 | Yes | 0.0026 | 0.0025 |
| DOX FCA NPs vs. MET&DOX FCA NPs | 0.2134 | No | 0.1216 | 0.2317 |

**Supplementary Table 7.** Ordinary one-way ANOVA Multiple comparisons (A375 cells Early apoptosis rate (%)).

| Two-stage linear step-up procedure of Benjamini, Krieger and Yekutieli | Mean Diff. | Discovery? | q value | Individual *P* Value |
| --- | --- | --- | --- | --- |
| CTL vs. MET FCA NPs | 1.14 | No | 0.4978 | 0.7111 |
| CTL vs. DOX FCA NPs | -9.32 | Yes | 0.0291 | 0.0139 |
| CTL vs. MET&DOX FCA NPs | -6.943 | Yes | 0.05 | 0.0476 |
| MET NPs vs. DOX FCA NPs | -10.46 | Yes | 0.0291 | 0.0078 |
| MET NPs vs. MET&DOX FCA NPs | -8.083 | Yes | 0.0367 | 0.0262 |
| DOX NPs vs. MET&DOX FCA NPs | 2.377 | No | 0.3753 | 0.4467 |

**Supplementary Table 8.** Ordinary one-way ANOVA Multiple comparisons (A375 cells Non-viable apoptosis and necrosis rat (%)).

| Two-stage linear step-up procedure of Benjamini, Krieger and Yekutieli | Mean Diff. | Discovery? | q value | Individual *P* Value |
| --- | --- | --- | --- | --- |
| CTL vs. MET FCA NPs | -19.91 | Yes | 0.0062 | 0.0297 |
| CTL vs. DOX FCA NPs | -23.31 | Yes | 0.0044 | 0.0149 |
| CTL vs. MET&DOX FCA NPs | -46.06 | Yes | 0.0003 | 0.0003 |
| MET NPs vs. DOX FCA NPs | -3.393 | No | 0.1163 | 0.6648 |
| MET NPs vs. MET&DOX FCA NPs | -26.15 | Yes | 0.0044 | 0.0085 |
| DOX NPs vs. MET&DOX FCA NPs | -22.76 | Yes | 0.0044 | 0.0166 |

**Supplementary Table 9.** Ordinary one-way ANOVA Multiple comparisons (SK-MEL-28 cells Early apoptosis rate (%)).

| Two-stage linear step-up procedure of Benjamini, Krieger and Yekutieli | Mean Diff. | Discovery? | q value | Individual *P* Value |
| --- | --- | --- | --- | --- |
| CTL vs. MET FCA NPs | -31.02 | Yes | <0.0001 | <0.0001 |
| CTL vs. DOX FCA NPs | -48.02 | Yes | <0.0001 | <0.0001 |
| CTL vs. MET&DOX FCA NPs | -61.99 | Yes | <0.0001 | <0.0001 |
| MET NPs vs. DOX FCA NPs | -17 | Yes | <0.0001 | <0.0001 |
| MET NPs vs. MET&DOX FCA NPs | -30.97 | Yes | <0.0001 | <0.0001 |
| DOX NPs vs. MET&DOX FCA NPs | -13.97 | Yes | <0.0001 | <0.0001 |

**Supplementary Table 10.** Ordinary one-way ANOVA Multiple comparisons (SK-MEL-28 cells Non-viable apoptosis and necrosis rate (%)).

| Two-stage linear step-up procedure of Benjamini, Krieger and Yekutieli | Mean Diff. | Discovery? | q value | Individual *P* Value |
| --- | --- | --- | --- | --- |
| CTL vs. MET FCA NPs | -10.85 | Yes | <0.0001 | <0.0001 |
| CTL vs. DOX FCA NPs | -16.31 | Yes | <0.0001 | <0.0001 |
| CTL vs. MET&DOX FCA NPs | -8.26 | Yes | <0.0001 | <0.0001 |
| MET NPs vs. DOX FCA NPs | -5.46 | Yes | <0.0001 | <0.0001 |
| MET NPs vs. MET&DOX FCA NPs | 2.587 | Yes | <0.0001 | <0.0001 |
| DOX NPs vs. MET&DOX FCA NPs | 8.047 | Yes | <0.0001 | <0.0001 |
